# Clinicopathological Pattern of Endometrial Specimens in Women with Abnormal Uterine Bleeding and Ultrasonography Correlation

**DOI:** 10.34172/aim.2024.31

**Published:** 2024-04-01

**Authors:** Mohadeseh Karimi, Anahita Alizadeh, Masoumeh Mahmoodi

**Affiliations:** ^1^Department of Pathology, Faculty of Medicine, Hormozgan University of Medical Sciences, Bandar Abbas, Iran; ^2^Department of Biostatistics, Hormozgan University of Medical Sciences, Bandar Abbas, Iran

**Keywords:** Abnormal uterine bleeding, Endometrium, Transvaginal sonography

## Abstract

**Background:**

Abnormal uterine bleeding (AUB) refers to any symptomatic deviation from normal menstruation. AUB is a common gynecological disorder in non-pregnant women of reproductive age, accounting for approximately 33% of gynecological outpatient visits. The early diagnosis and management cause of AUB is important because of increased incidence of endometrial carcinoma with rapid growth. Transvaginal ultrasound is non-invasive imaging technique used to find endometrial carcinoma before referring patients for invasive techniques. Dilatation and curettage (D&C) and endometrial biopsy are surgical procedures that scrape the endometrial lining of the uterus for diagnosis and treatment. The aim of this study is to describe the clinicopathologic pattern of endometrial specimens in women with AUB and ultrasonographic correlation.

**Methods:**

Tissues from endometrial biopsy and curettage of 411 patients with AUB who referred to Shahid Mohammadi hospital were prospectively selected from 2021 to 2023. Patients were divided into three groups based on age and menstrual status including: premenopausal (18-39 years), perimenopausal (40-49 years) and postmenopausal (≥50 years). The results were correlated to patient’s age and other data and evaluated with statistical analysis.

**Results:**

During the two-year study period, a total of 411 endometrial specimens with clinical diagnosis of AUB were submitted and the results were analyzed. The youngest patient presenting with AUB was 21 years old, while the oldest was 77 years old. The most common complaint was menorrhagia in 201 (48.0%) out of 411 patients. The most common pathology finding in three groups was polyp in 100 (24.3%) cases. Hormonal effect was the next commonly observed pattern seen in 70 (17.0%) cases. P value was calculated as 0.003 which was significant using chi-square for the trend seen in age.

**Conclusion:**

Endometrial sampling is a useful tool for evaluation of women with AUB and referring patients for treatment. Histopathological evaluation of the endometrium is very useful in detecting the etiology of AUB. Transvaginal sonography has high sensitivity in detecting polyps.

## Introduction

 In a normal menstrual cycle, with an interval of 28 ± 7 days and duration of 4 ± 3 days, blood loss is 30 to 80 mL/cycle. Abnormal uterine bleeding (AUB) refers to any symptomatic deviation of the following parameters from normal menstruation: frequency, regularity, duration, and volume. AUB includes various features and types such as oligomenorrhea, menorrhagia, menometrorrhagia, polymenorrhagia and spotting.^1,2^ In postmenopausal women, it is defined as any bleeding one year after menstruation stops.^3^ AUB is a common gynecological disorder in non-pregnant women of reproductive age, accounting for approximately 33% of gynecological outpatient visits.^4^ It may have a significant effect on women’s physical, social, emotional and material quality of life.^5^ In addition to the direct impact on women and their families, there is a huge cost to the economy and health services.^6^

 The International Federation of Gynecology and Obstetrics (FIGO) classifies the etiology of AUB into 9 categories with a developed acronym PALM-COEIN. “PALM” refers to structural causes: Polyps; Adenomyosis; Leiomyomas; and Malignancy/Hyperplasia and “COEIN” refers to non-structural causes: Coagulopathy; Ovulatory dysfunction; Endometrial causes; Iatrogenic causes; Not Otherwise classified.^7^ In addition, long-term use of progestin-only contraceptives is associated with abnormal bleeding due to adverse effects on the endometrial microvasculature.^8-10^

 Early diagnosis and management of the cause of AUB is important because the increased incidence of endometrial carcinoma with rapid growth is expected to reach 50% by 2040.^11^

 Different diagnostic modalities can be used to diagnose the cause of AUB.^12^ Transvaginal ultrasound is a non-invasive imaging technique used to evaluate endometrial thickness, a marker and screening tool for endometrial carcinoma before referring patients for invasive techniques such as endometrial biopsy by dilatation and curettage (D&C).^13^ D&C and endometrial biopsy are important tools in diagnosis and treatment of AUB cases.^14^ D&C is a surgical procedure to scrape the endometrial lining of the uterus for diagnosis and treatment.^15^ Endometrial sampling is recommended for all women in perimenopausal age and above; especially, those at risk of atypical hyperplasia or carcinoma patients are selected for endometrial sampling to detect any histopathological atypia.^16,17^

 The aim of this study is to describe the prevalence of different histopathological findings in endometrial specimens of AUB cases who underwent this procedure in our hospital over a 2-year period. We further compared histopathology findings with ultrasonography findings and clinical reports.

## Materials and Methods

 Using the formula Z21-alpha/2 pq/d2 = 3.84*0.25*0.75/0.0025 = 288 and taking into account the first type error of 5% and the precision of 5% and the information taken from the study by Bajithak et al, a sample size of 288 was calculated. In our study, tissues from endometrial biopsy and curettage of 411 patients with AUB who referred to Shahid Mohammadi hospital were prospectively selected from 2021 to 2023. An informed consent form was completed for each patient. The patient’s data including age, bleeding duration, menstruation period status and history of hormonal pill consumption, as well as para-clinical data including vaginal or pelvic ultrasonography and clinical reports were recorded. Patients were divided into three groups based on age and menstrual status including: premenopausal (18-39 years), perimenopausal (40-49 years) and postmenopausal (≥ 50 years). Endometrial specimens were processed and H&E slides were prepared and underwent microscopic examination by an expert pathologist. The results were correlated to the patient’s age and other data and evaluated with statistical analysis using SPSS (version 19) for Windows. The percentages of each pathologic condition in each age group and possible leading cause were reported. A *P* value less than 0.003 was considered significant. P values were calculated using F test.

## Results

 During the two-year study period, a total of 411 endometrial specimens with clinical diagnosis of AUB were submitted and the results were analyzed. The youngest patient presenting with AUB was 21 years old, while the oldest patient was 77 years old. Most patients were in the perimenopausal age group of 40-49 years, followed by the premenopausal age group of 21-39 years. Analysis of the distribution of births showed that most of women were multiparous (58.0%) and 7.0% of cases were nulliparous. The contraceptive methods used in the premenopausal and perimenopausal age groups were OCP in 90, condom in 100, IUD in 40, TL in 15, and natural in 73 cases.

 The most common complaint was menorrhagia in 201 (48.0%) of 411 patients. The most common pathology finding in the study was polyp in 100 (24.3%) cases. Hormonal effect was the next most commonly observed pattern seen in 70 (17.0%) cases. Proliferative endometrium and inadequate sampling were reported in 53 (12.9%) and 48 (11.7%) cases, respectively. Secretory endometrium and disordered proliferative endometrium were found in 35 (8.5%) cases each. Endometrial hyperplasia was observed in 30 (7.3%) cases. Menstrual endometrium was found in 18 (4.4%) cases, and finally, menstrual anovulatory endometrium was seen in 11 (2.7%) cases (Table 1).

**Table 1:** Demographic and Clinical Characteristics by Age Group (Main Variable)

**Variables**	**Perimenopausal No. (%)**	**Postmenopausal No. (%)**	**Premenopausal No. (%)**	**Total No. (%)**	***P *** **Value**
Secretory endometrium	14 (3.4)	6 (1.5)	15 (3.6)	35 (8.5)	0.003
Menstrual anovulatory endometrium	7 (1.7)	4 (1.0)	0 (0.0)	11 (2.7)	
Endometrial hyperplasia	12 (2.9)	11 (2.7)	7 (1.7)	30 (7.3)	
Polyp	41 (10.0)	20 (4.9)	39 (9.5)	100 (24.3)	
Hormonal effect	33 (8.0)	8 (2.0)	29 (7.0)	70 (17.0)	
Proliferative endometrium	19 (4.6)	6 (1.5)	23 (5.6)	48 (11.7)	
Endometrial carcinoma	0 (0.0)	3 (0.7)	0 (0.0)	3 (0.7)	
total	126 (30.6)	58 (14.3)	113 (27.4)	297 (72.2)	

 Chronic endometritis was found in 5 (1.2%) cases. Endometrial carcinoma was seen in 3 (0.7%) cases. Atrophic endometrium was found in 2 (0.5%) cases and submucosal leiomyoma was seen in 1 (0.2%) case (Table 2).

**Table 2:** Demographic and Clinical Characteristics by Age Group (Sub Variable).

**Variables**	**Perimenopausal No. (%)**	**Postmenopausal No. (%)**	**Premenopausal No. (%)**	**Total No. (%)**	***P *** **Value**
Inadequate sample	21 (5.1)	21 (5.1)	11 (2.7)	53 (12.9)	0.003
Disordered proliferative endometrium	13 (3.2)	8 (1.9)	14 (3.4)	35 (8.5)	
Shedding endometrium	8 (1.9)	4 (1.0)	6 (1.5)	18 (4.4)	
Atrophic endometrium	1 (0.2)	1 (0.2)	0 (0.0)	2 (0.5)	
Submucosal leiomyoma	1 (0.2)	0 (0.0)	0 (0.0)	1 (0.2)	
Chronic endometritis	1 (0.2)	1 (0.2)	3 (0.7)	5 (1.2)	
Total	45 (10.8)	35 (8.4)	34 (5.6)	114 (27.8)	

 Polyp and proliferative endometrium were the most common patterns seen in the premenopausal group. In the perimenopausal group, polyp was the most common followed by hormonal effect. In the postmenopausal group, inadequate sampling was the most common pattern followed by polyp. Most of the endometrial and other carcinomas were found in the postmenopausal group (Table 1).

 The most common abnormality reported by transvaginal sonography was thickened endometrium (46%), followed by endometrial polyp (27%), submucous myoma (10%), adenomyosis (2%), and malignancy (1%) (Table 3).

**Table 3:** Causes of AUB Identified by Transvaginal Sonography

**Findings**	**Number of Cases**	**Percent**
Thickened endometrium	190	46
Endometrial polyp	112	27
Submucosal leiomyoma	41	10
adenomyosis	8	2
malignancy	4	1
Ovary cyst	12	3
No abnormal findings	45	11

Table 4 shows the overall sensitivity, specificity, positive predictive value (PPV) and negative predictive value (NPV) for transvaginal sonography in the diagnosis of uterine abnormalities. Transvaginal ultrasound had high sensitivity in detecting polyps. However, in leiomyoma, transvaginal ultrasound findings do not match histologically because the sample of endometrial biopsy and curettage is almost from the mucosal surface and not from the muscle layer.

**Table 4:** Diagnostic Accuracy of TVS in AUB

**Findings**	**Sensitivity (95% CI)**	**Specificity (95% CI)**	**PPV (95% CI)**	**NPV (95%CI)**
Thickened Endometrium	82% (75.61% to 88.52%)	23% (8.22% to 47.17%)	64% (59.38% to 65.32%)	25% (10.92% to 37.75%)
Polyp	89% (83.68% to 92.51%)	47% (28.34% to 65.67%)	77% (73.63% to 79.47%)	28% (22.76% to 37.32%)
Submucosal leiomyoma	5% (0.63% to 17.32%)	70% (57.10% to 82.37%)	8% (2.96% to 33.90%)	53% (46.71% to 55.90%)
Malignancy	72% (61.78% to 81.15%)	57% (40.82% to 73.69%)	55% (47.63% to 61.32%)	67% (56.63% to 78.32%)

 Microscopic examination of endometrial polyp, endometrial hyperplasia, endometrioid carcinoma, proliferative phase, secretory phase, menstrual phase and atrophic phase of endometrium showed various histological findings (Figures 1-9).

**Figure 1:**
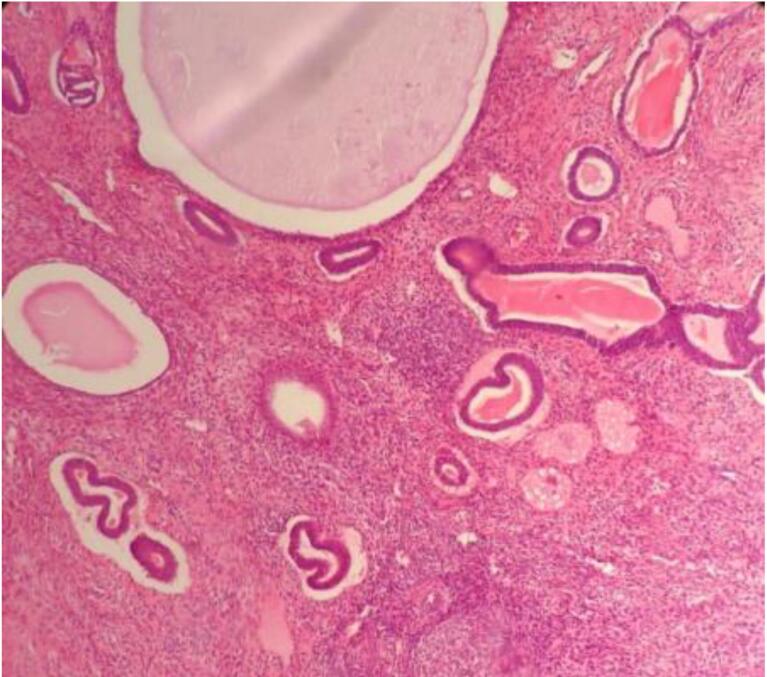
Polyp: the photomicrograph shows cystically dilated glands along with compact stroma and thick wall blood vessels. Hematoxylin and eosin (H&E) stain (× 400)

**Figure 2:**
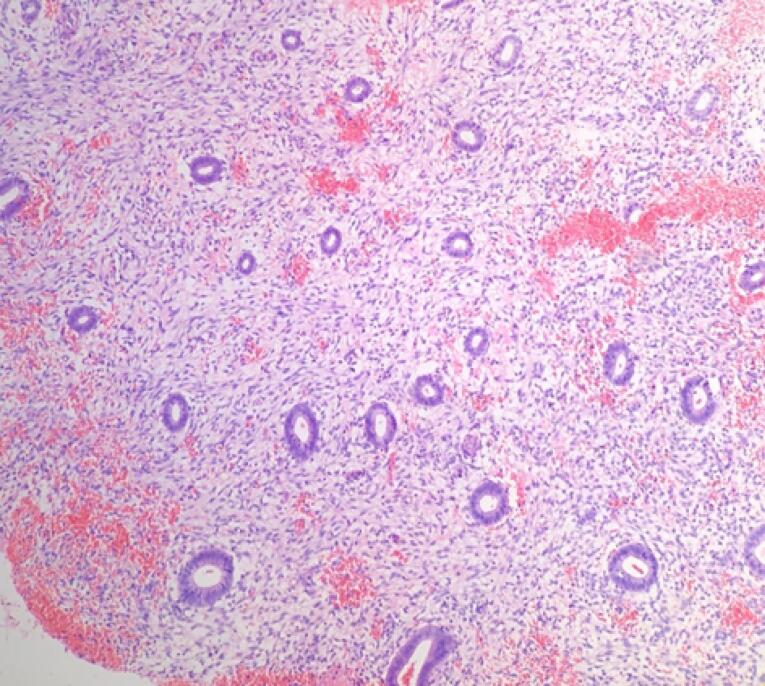
Hormonal Effect: The photomicrograph shows small glands with abundant decidua. Hematoxylin and eosin (H&E) stain (× 400)

**Figure 3:**
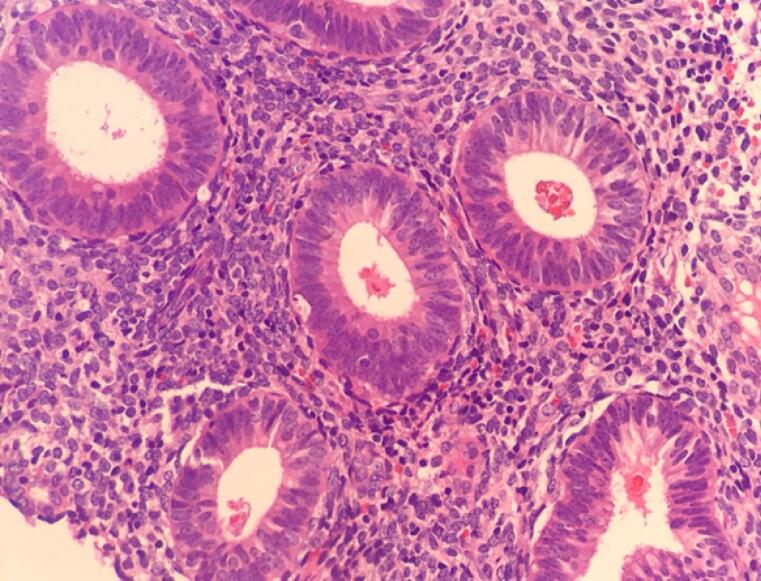
Proliferative Endometrium: The photomicrograph shows uniform small glands along with compact stroma. Hematoxylin and eosin (H&E) stain (× 400)

**Figure 4:**
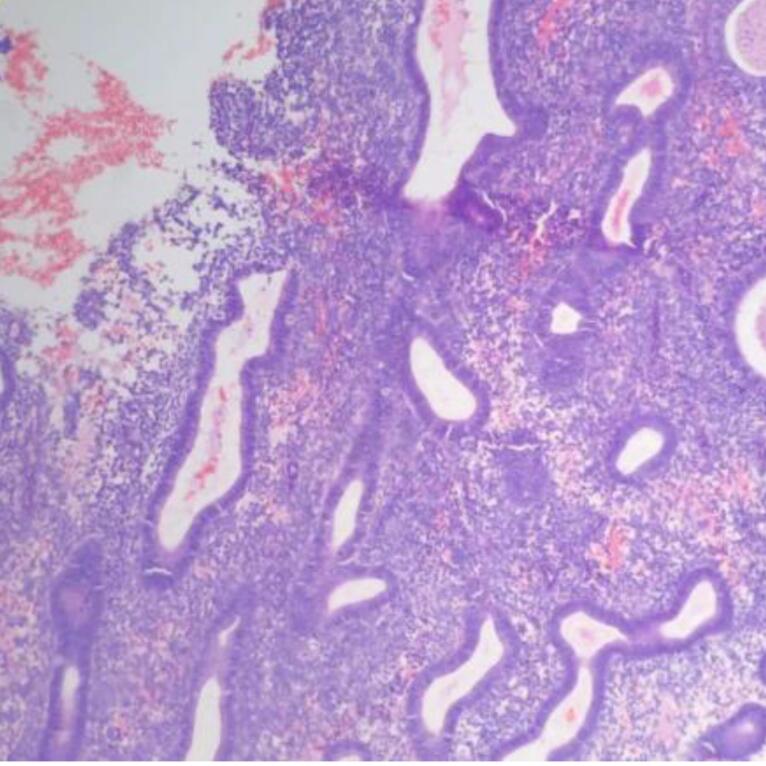
Disordered Proliferative Endometrium: The photomicrograph shows disordered glandular proliferation with variably sized and shaped glands. Hematoxylin and eosin (H&E) stain (× 400).

**Figure 5:**
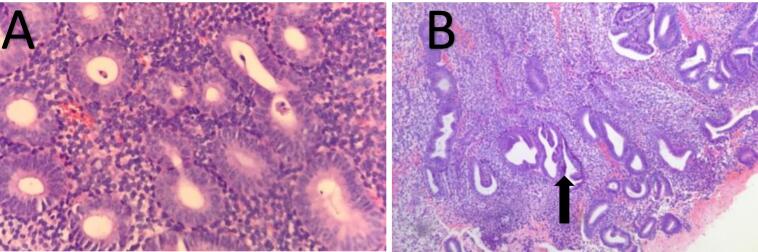
Endometrial Hyperplasia: A) Glandular Proliferation in Compact Stroma; B) Crowded Glands with Branching. Hematoxylin and eosin (H&E) stain (× 400) (see black arrows)

**Figure 6:**
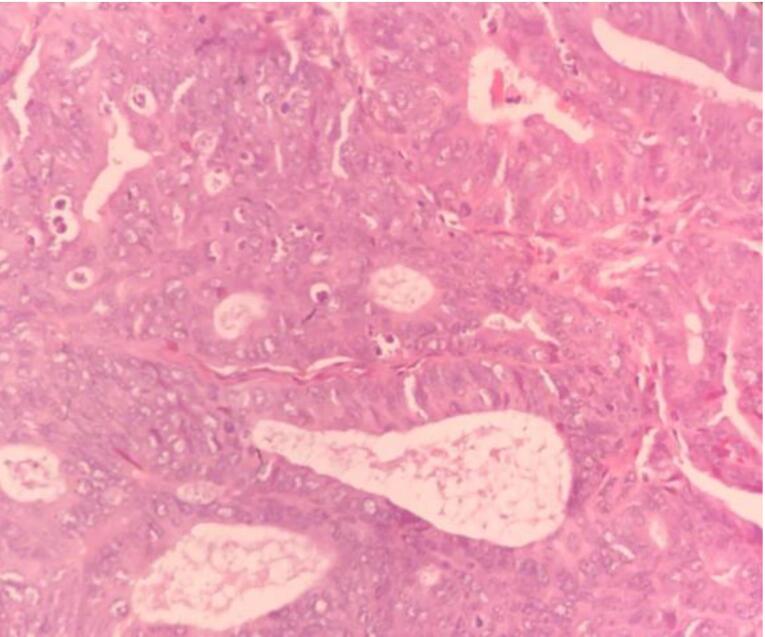
Endometroid Carcinoma: The photomicrograph shows the neoplastic glands with back to back arrangement and nuclear pleomorphism, hyperchromatism and prominent nucleoli of epithelial lining. Hematoxylin and eosin (H&E) stain (× 400)

**Figure 7:**
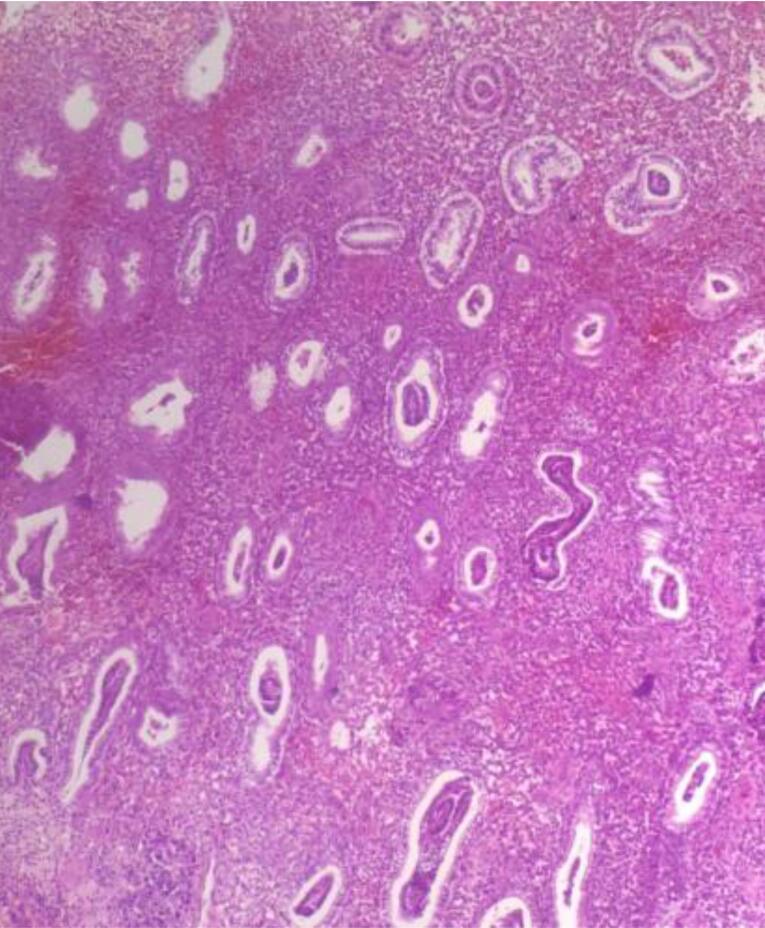
Secretory Endometrium: The photomicrograph shows subnuclear vacuolation of glands along with mild stromal edema. Hematoxylin and eosin (H&E) stain (× 400)

**Figure 8:**
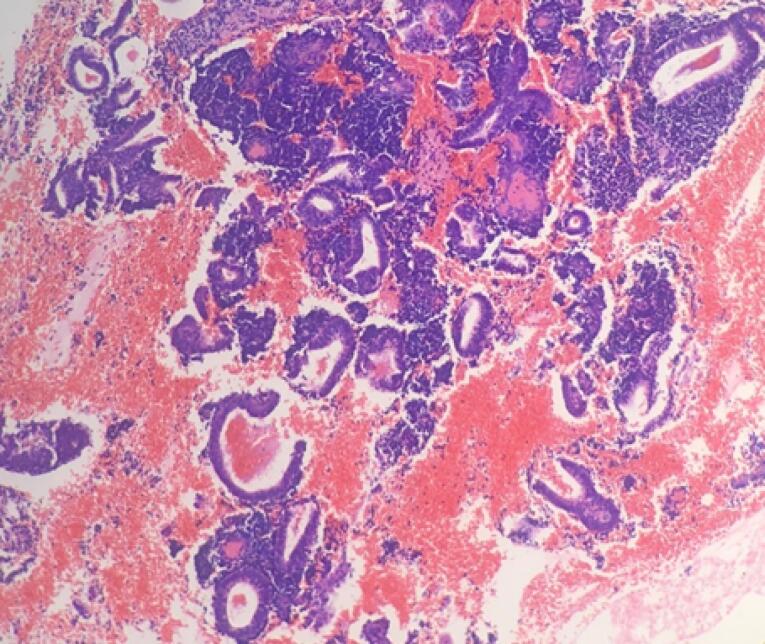
Menstrual Endometrium: The photomicrograph shows broken and fragmented endometrial glands. Hematoxylin and eosin (H&E) stain (× 400)

**Figure 9:**
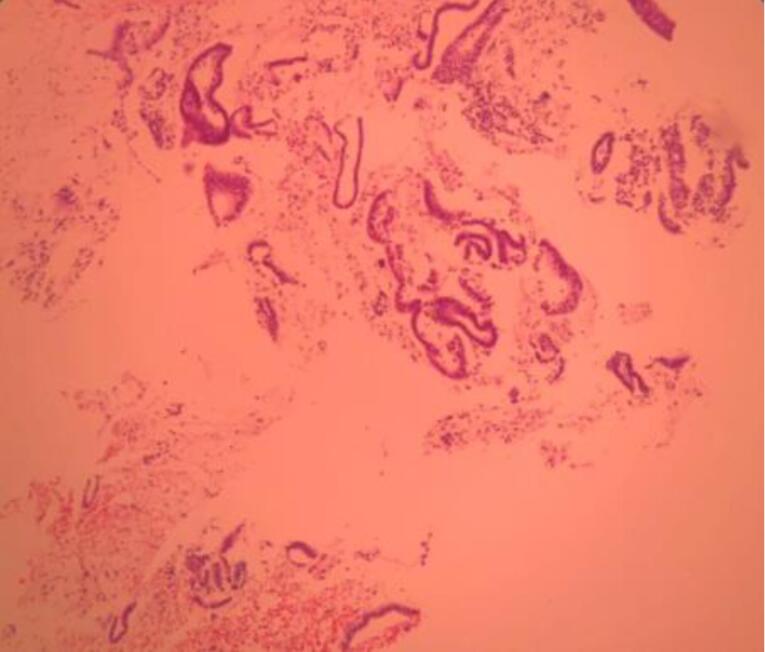
Atrophic Endometrium: The photomicrograph shows multiples strips of mitotically inactive endometrial glands with flattened epithelium. Hematoxylin and eosin (H&E) stain (× 400)

## Discussion

 In this study, perimenopausal women constituted the most common age group for AUB (171 patients), followed by premenopausal women (147 patients). Other studies have found that AUB is most commonly found in perimenopausal women.^18-24^ Our study, and others, found the most common complaint of patient to be menorrhagia.^18-22^

 In this study, preoperative transvaginal sonography was done to identify the cause of AUB. Transvaginal sonography is a practical and initial way to detect possible reasons for AUB. The findings of transvaginal sonography are shown in Table 3. The most common pathology finding in our study was polyps in 100 (24.3%) cases. Preoperative transvaginal sonography has high sensitivity for polyp detection.

 The prevalence of polyps was reported in studies done by Lairah et al (40%).^25^ In a study by Dhruvi et al, endometrial polyps were found in 2% of women.^1^ A polyp is a focal hyperplastic overgrowth of endometrial glands and stroma around a vascular core in the uterine cavity.^26^ Possible etiologies include genetic, biochemical, and hormonal factors.^27,28^ In our study, it was related to OCP use in 17 (4.0%) patients.

 Hormonal effect was the second most common histopathological finding identified in 75 women (18.2%) that was comparable with the study done by Alshdaifat et al (16.9%).^3^

 We found endometrial hyperplasia in 30 (7.3%) women, most commonly in the perimenopausal age group (2.9%). In other studies, endometrial hyperplasia was found most commonly in the postmenopausal age group.^29-35^ Prolonged endogenous (chronic anovulation) or exogenous (hormone replacement therapy) estrogen can stimulate the glandular and stromal overgrowth that causes endometrial hyperplasia. Early diagnosis of endometrial hyperplasia is important as it may precede or coexist with endometrial cancer.^36^ Therefore, in the perimenopausal age group with AUB, any dangerous pathology findings should always be ruled out.

 In our study, endometrial carcinoma was found in 3 (0.7%) women. This was comparable with the findings reported by Shah et al (0.3%),^37^ Gulia et al (1%),^29^ Vaidyea et al (1%),^30^ Mishra et al (2%)^38^ and Khan et al (2%).^33^

 In this study, the mean age of patients who presented with postmenopausal bleeding was 62 years. The histological type of malignancy was endometrioid adenocarcinoma.

 Previous studies have reported different incidence rates of endometrial carcinoma due to the variation in the geographical location, lifestyle, socioeconomic conditions, as well as their inclusion criteria.^1^

 We found chronic endometritis in 5 (1.2%) women, most commonly seen in the premenopausal age group (0.7%), due to abortions, intra-uterine contraceptive devices, and ascending route of infections.

 In the present study, 53 (12.9%) samples were insufficient for histopathological diagnosis. In a study by Alshdaifat et al, inadequate sampling was reported in 101 (51.8%) women.^3^

 Endometrial sampling techniques used in our study were Pipelle and D&C. Pipelle causes less pain, less tissue volume and is more cost effective compared with D&C.^39^ In our study, inadequate sampling was most commonly seen with Pipelle (65%).

 Endometrial sampling is a useful tool for evaluation of women with AUB and referring patients for treatment. Histopathological evaluation of the endometrium is very useful in detecting the etiology of AUB. Our histopathological analysis showed that the most common pattern in endometrial specimens of AUB cases was polyps, followed by hormonal effect and proliferative endometrium. Transvaginal sonography has high sensitivity in detecting polyps.
